# Consolidating biallelic *SDHD* variants as a cause of mitochondrial complex II deficiency

**DOI:** 10.1038/s41431-021-00887-w

**Published:** 2021-05-20

**Authors:** Siying Lin, James Fasham, Fida’ Al-Hijawi, Nouar Qutob, Adam Gunning, Joseph S. Leslie, Lucy McGavin, Nishanka Ubeyratna, Wisam Baker, Ramez Zeid, Peter D. Turnpenny, Andrew H. Crosby, Emma L. Baple, Reham Khalaf-Nazzal

**Affiliations:** 1 RILD Wellcome Wolfson Centre, University of Exeter Medical School, Royal Devon & Exeter NHS Foundation Trust, Exeter, UK; 2 Peninsula Clinical Genetics, Royal Devon & Exeter Hospital (Heavitree), Exeter, UK; 3 Paediatrics’ Community Outpatient Clinics, Palestinian Ministry of Health, Jenin, Palestine; 4 Department of Health Sciences, Faculty of Graduate Studies, Arab American University of Palestine, Ramallah, Palestine; 5 University Hospitals Plymouth NHS Trust, Plymouth, UK; 6 Paediatrics Department, Dr. Khalil Suleiman Government Hospital, Jenin, Palestine; 7 Biomedical Sciences Department, Faculty of Medicine, Arab American University of Palestine, Jenin, Palestine

**Keywords:** Disease genetics, Genetic counselling, Metabolic disorders, Genetics research

## Abstract

Isolated mitochondrial complex II deficiency is a rare cause of mitochondrial respiratory chain disease. To date biallelic variants in three genes encoding mitochondrial complex II molecular components have been unequivocally associated with mitochondrial disease (*SDHA/SDHB/SDHAF1*). Additionally, variants in one further complex II component (*SDHD*) have been identified as a candidate cause of isolated mitochondrial complex II deficiency in just two unrelated affected individuals with clinical features consistent with mitochondrial disease, including progressive encephalomyopathy and lethal infantile cardiomyopathy. We present clinical and genomic investigations in four individuals from an extended Palestinian family with clinical features consistent with an autosomal recessive mitochondrial complex II deficiency, in which our genomic studies identified a homozygous NM_003002.3:c.[205 G > A];[205 G > A];p.[(Glu69Lys)];[(Glu69Lys)] *SDHD* variant as the likely cause. Reviewing previously published cases, these findings consolidate disruption of SDHD function as a cause of mitochondrial complex II deficiency and further define the phenotypic spectrum associated with *SDHD* gene variants.

## Introduction

The mitochondrial oxidative phosphorylation (OXPHOS) system is composed of five multi-subunit transmembrane protein complexes (I–V) encoded by the mitochondrial and nuclear genomes, and is the primary mechanism for adenosine triphosphate production in eukaryotic cells. OXPHOS defects result in mitochondrial disease, with an estimated prevalence of 1:4300 [1, 2].

Mitochondrial complex II (succinate dehydrogenase) is composed of two catalytic subunits (SDHA/SDHB) anchored to the inner mitochondrial membrane by two smaller subunits (SDHC/SDHD) [3, 4]. Complex II differs from other mitochondrial respiratory chain complexes, in that the four structural subunits and their two assembly factors (SDHAF1/SDHAF2) are solely encoded by the nuclear genome. Complex II is also unique in being involved in both the mitochondrial respiratory chain and the Krebs cycle [2].

Mitochondrial complex II deficiency with multisystem involvement has been reported in association with biallelic *SDHA* [5], *SDHB* [2], *SDHD* [6, 7] and *SDHAF1* [3, 8] gene variants, with clinical presentations including Leigh syndrome, leukoencephalopathy, optic atrophy and cardiomyopathy with highly variable severity and age of onset [5, 9]. Complex II deficiency is rare accounting for only 2–4% of OXPHOS defects [6], with variants in *SDHA* being most common, predominantly associated with Leigh syndrome [5]. Previously, only two individuals with candidate biallelic *SDHD* variants and isolated complex II deficiency have been reported [6, 7]. Here we describe four Palestinian siblings presenting in childhood with clinical features indicative of mitochondrial disease and a likely pathogenic homozygous *SDHD* variant, consolidating *SDHD* gene variants as a likely cause of autosomal recessive mitochondrial complex II deficiency.

## Materials and methods

Blood samples were collected with informed consent (Palestinian Health Research Council; PHRC/HC/518/19). Single-nucleotide polymorphism (SNP) genotyping was performed (HumanCytoSNP-12 v2.1 Beadchip array: Illumina). Whole-exome sequencing (WES) (NextSeq1500: Illumina) analysis involved: Agilent SureSelect Whole Exome v6 targeting, read alignment (BWA-MEM,v0.7.17), mate-pairs fixed and duplicates removed (Picard v2.15.0), InDel realignment and base quality recalibration (GATK v3.7.0), SNVs/InDels (GATK/HaplotypeCaller), annotation using Alamut Batch (v1.10) and CNV detection with ExomeDepth [10] and Savvy CNV [11]. Dideoxy sequencing was undertaken using standard techniques.

Model 3abv (porcine heart mitochondrial complex II) [12] was selected from X-ray diffraction/NMR-derived structures of SDHD (O14521) and its homologues (RCSB Protein Data Bank). Amino acid residues were visualised with their polar bonds, and annotated using Pymol 2.3 [13, 14].

## Results

### Clinical findings

**Fig. 1:**
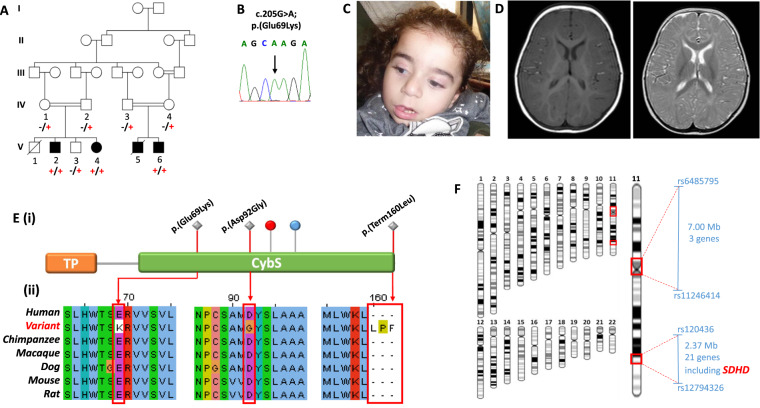
Family pedigree showing *SDHD* c.205 G > A genotype data, neuroimaging and images of affected individuals. **A** Pedigree diagram showing segregation of the *SDHD* c.205 G > A; p.(Glu69Lys) variant. Genotypes are shown beneath generations IV and V (+, c.205 G > A; −, WT). Affected individuals were homozygous for SDHD c.205 G > A, DNA was available from all but one affected individual (V:5). **B** Electropherogram showing the DNA sequence at the position of *SDHD* c.205 G > A in a homozygous affected individual. **C** T1- and T2-weighted axial views of MRI head of individual V:2 (aged 8 months). Normal myelination and no intracranial abnormalities. **D** Image of affected individual V:4, illustrating the absence of any facial dysmorphism. **E** (i) Schematic localisation of *SDHD* p.(Glu69Lys), p.(Asp92Gly) and p.(Ter160LeuextTer3) variants within the succinate dehydrogenase cytochrome b small subunit (CybS) domain of the SDHD polypeptide. The orange rectangle denotes the transit peptide (TP) domain, the red circle denotes the iron (haem axial ligand) binding site shared with SDHC and the blue circle denotes the ubiquinone binding site shared with SDHB. (ii) Conservation of the SDHD p.(Glu69Lys), p.(Asp92Gly) and p.(Ter160LeuextTer3) variants across species. **F** Visual depiction of the two autozygous regions on chromosome 11 (shown in red) common to affected individuals V:2, V:4 and V:6 including the 2.37 Mb region containing 21 genes including *SDHD*.

We describe four affected Palestinian patients (three male, one female) aged 4–20 years, comprising of two sibships from an extended interconnecting family (Fig. 1A). All four children presented with developmental delay in infancy and variable clinical and laboratory findings suggestive of a mitochondrial disorder including elevated serum lactate/urinary Krebs cycle metabolites, nystagmus, optic atrophy, progressive microcephaly, generalised hypotonia, epileptic seizures, severe/profound intellectual disability/developmental impairment and cardiomyopathy. The affected children were not dysmorphic (Fig. 1C), though individuals V:2 and V:4 were noted to have significant hypertrichosis, particularly over their back and limbs. MRI neuroimaging was unremarkable for one child at 8 months (V:2; Fig. 1D), however, his sister’s scan revealed delayed myelination at age 6 months (V:4). Hirschsprung disease, confirmed by aganglionic rectal biopsy, was noted in a single individual (V:2). A full description of the clinical features and disease progression is summarised in Table 1.

**Table 1:** Clinical features of affected individuals with mitochondrial complex II deficiency due to biallelic *SDHD* variants.

	Jackson et al.	Alston et al.	This study; V:2		This study; V:4	This study; V:6	This study; V:5
Genotype (NM_003002.3)	p.[(Glu69Lys)]; [(Ter160LeuextTer3)]	p.[(Asp92Gly)]; [(Asp92Gly)]	p.[(Glu69Lys)]; [(Glu69Lys)]		p.[(Glu69Lys)]; [(Glu69Lys)]	p.[(Glu69Lys)]; [(Glu69Lys)]	NA (deceased)
Ethnicity	Swiss	Irish	Palestinian		Palestinian	Palestinian	Palestinian
Gender	F	M	M		F	M	M
Age at last evaluation	7 yrs (deceased age 10 yrs)	Deceased day 1 of life from lethal cardiomyopathy	6.4 yrs		4.5 yrs	20 yrs	Deceased age 10 yrs—cardiac arrest
Growth parameters							
Birth weight kg (SDS)	NA	2.62	3.5 (−0.1)		2.8 (−1.4)	NA	NA
Birth OFC cm (SDS)	NA	34.5	35 (−0.2)		35 (+0.4)	NA	NA
OFC cm (SDS)	2° microcephaly from 2 yrs	NA	46.5 (−4.4)		49 (−2.0)	NA	NA
Development							
Intellectual disability	Severe Maximum developmental age of 11 mo at 4 yrs	NA	Profound		Profound	Severe	Severe
Developmental regression	✓ (from age 3 mo after bronchiolitis) Several subsequent episodes of regression after infection/prolonged fasting	NA	✓ (from age 5 mo following surgery) Previously was sitting with support, mouthing, purposeful hand movements		✓ (from age 4 mo) Previously sitting with support, fixing and following, mouthing, purposeful hand movements	No clear hx of regression	No clear hx of regression
Gross motor skills	NA	NA	Antigravity movements of arms and legs only		Sits with support. Rolls from back to front	Sits with support	Walked with support
Fine motor skills	NA	NA	No active hand use		No active hand use	Finger feed	Finger fed
Expressive and receptive language development	NA	NA	Vocalisation, makes sucking motions if thirsty		Vocalisation, responds to loud noises	Vocalisation, points to indicate needs	2 word phrases
Behavioural abnormalities	NA	NA	Sleep disturbances treated with risperidone		None	Repetitive hand movements	_
Neurology							
Generalised muscle hypotonia	✓	NA	✓	✓		✓	NK
Movement disorder	Dystonia and ataxia	NA	NA	NA		Dystonia	
Seizures	✓ Polymorphic epilepsy and intractable myoclonic movements	NA	Generalised seizures post surgery (6 mo)	Abnormal movements (4 mo)		Seizures when younger now resolved	_
EEG	Normal	NA	Normal (7 mo)	Normal		NK	NK
Neuroimaging	Normal MRI (10 mo and 2 yrs)	NA	Normal MRI (8 mo)	MRI: delayed myelination (6 mo)		Normal CT brain (7 yrs)	NK
Ocular							
Visual impairment	✓	NK	✓	✓		✓	NK
Nystagmus	✓	NK	✓	✓		✓	✓
Optic atrophy	✓	NK	✓	✓		✓	NK
Strabismus	NA	NK	NK	✗		✓	✓
Hearing impairment	✗	NK	✗	✗		✗	✗
Cardiac abnormalities	NK	**✓** Hypertrophic cardiomyopathy with Lt ventricular non-compaction (prenatal onset)	✗ Normal cardiac structure and function (7 yrs)	✗ Normal cardiac structure and function (2.8 yrs)		✓ Minimal left ventricular hypertrophy, with low normal Lt ventricular function (21 yrs)	✓ Echo (5 yrs): dilated cardiomyopathy with reduced left ventricular ejection fraction, mild mitral and tricuspid regurgitation
Hypertrichosis	NK	NK	✓	✓		✗	NK
Metabolic investigations	Raised serum lactate (10.2 mmol/L), lactic aciduria and ketonuria, urinary Krebs cycle intermediates Marked defect in complex II activity in muscle homogenate	Marked defect in complex II activity in muscle homogenate	None	Raised serum lactate (5.58 mmol/L) Urinary excretion of Krebs cycle metabolites (succinic, fumaric and ketoglutaric acids)		Normal respiratory chain complexes II-IV in fibroblast homogenate (succinate: cytochrome c reductase assay was outside the normal range, but reported as normal) Non-specific muscle biopsy findings	NK
Other clinical features	–	–	Hirschsprung disease diagnosed at 1 mo, Frequent LRTI	–		–	–

The (✓) and (✗) symbols indicate the presence or absence of a feature in an affected subject, respectively. Height, weight and OFC Z-scores were calculated using a Microsoft Excel add-in to access growth references based on the LMS method^1^ using a reference European population^2^.

*CT* computerised tomography, *Echo* Echocardiogram, *EEG* electroencephalogram, *F* female, *hx* history, *Lt* left, *LRTI* lower respiratory tract infection, *M* male, *mo* months, *MRI* magnetic resonance imaging, *NA* not available, *NK* not known, *OFC* occipitofrontal circumference, *SDS* standard deviation scores, *yr* years.

Supplementary references: 1. Pan H, Cole TJ. LMS growth, a Microsoft Excel add-in to access growth references based on the LMS method. Version 2.77. http://www.healthforallchildren.co.uk/;2012. 2. Cole TJ, Freeman JV, Preece MA. British 1990 growth reference centiles for weight, height, body mass index and head circumference fitted by maximum penalized likelihood. Stat Med. 1998;17:407–29.

### Genetic findings and homology modelling

Genome-wide SNP genotyping and WES were undertaken assuming that a homozygous founder variant was responsible, although also considering other genetic mechanisms. SNP genotyping (individuals V:2, V:4 and V:6) identified four notable (>1 Mb) shared homozygous regions, the two largest identified on chromosome 11; a ~7.00 Mb region (rs6485795–rs11246414, Chr11:g.47908294–54905443 [hg38]) and a ~2.42 Mb region (rs120436–rs12794326, Chr11:g.110826521–113248134) (Fig. 1F). WES was performed on DNA from affected individual V:4, to identify rare functional candidate variants. Variants were prioritised by call quality and frequency (gnomAD v2.1.1/1000 Genomes Project, MAF ≤ 0.0001) and cross referenced with SNP data, identifying only a single candidate homozygous variant of relevance to the phenotype in *SDHD* NM_003002.3:c.[205G > A];[205G > A];p.[(Glu69Lys)];[(Glu69Lys)];Chr11:g.[112088902G > A];[112088902G > A], located within the second largest shared homozygous region. The variant is present in only two heterozygotes in gnomAD (v2.1.1) and is predicted to result in a glutamic acid—lysine substitution in an evolutionarily conserved Glu69 residue (Fig. 1E). This variant was previously reported as the likely candidate cause of disease in compound heterozygous form ((c.[205G > A];[479G > T]; p.[(Glu69Lys)];[(Ter160LeuextTer3)])) in a single individual with autosomal recessive encephalomyopathy and isolated mitochondrial complex II deficiency [6] (ClinVar accession:VCV000156153.8 and SCV001424558). Dideoxy sequencing confirmed cosegregation as appropriate for an autosomal recessive disorder (Fig. 1A, B ). Protein modelling positions the p.(Glu69Lys) substitution within the first transmembrane alpha helix, where it likely disrupts tertiary structure through interrupting a predicted hydrogen bond with Gln109 on the adjacent helix (Supplementary Fig. S1A). Conversely, a previously described variant, p.(Asp92Gly) is located at the apex of transmembrane alpha helices one and two, in close proximity to the membrane and inter-membrane space (Supplementary Fig. S1A, B).

## Discussion

Here we define a homozygous *SDHD* c.[205G > A];[205G > A];p.[(Glu69Lys)];[(Glu69Lys)] missense variant as the likely cause of isolated mitochondrial complex II deficiency in three affected children from an extended Palestinian family. DNA was unavailable for V:5 (deceased age 10 years), whose clinical history overlapped that of his sibling (V:6). Tissues and organs heavily dependent on robust OXPHOS processes tend to be most affected by mitochondrial disease [15], explaining why common findings include optic atrophy, leukoencephalopathy, myopathy, cardiomyopathy and Leigh syndrome. These clinical features overlap those described in the two individuals with SDHD-related mitochondrial disease reported to date (Table 1). Previously, compound heterozygous variants in *SDHD* [6], including the same p.(Glu69Lys) variant identified here and a c.479G > T; p.(Ter160LeuextTer3) alteration (c.[205G > A];[479G > T];p.[(Glu69Lys)];[(Ter160LeuextTer3)]), were identified as the likely candidate cause of disease in a Swiss child presenting with developmental regression following a viral infection, at 3 months. Progressive ocular (visual impairment, nystagmus, optic disc pallor) and neurological (epileptic seizures, ataxia, dystonia and continuous intractable myoclonic movement) involvement were described, and the child died aged 10 years. Urinalysis revealed lactic aciduria, ketonuria and Krebs cycle intermediates. Complex II activity was deficient in skeletal muscle and complementation studies in patient fibroblasts showed restoration of complex II assembly and function with expression of wildtype, but not mutant, *SDHD* cDNA [6]. Subsequently an Irish male infant was described [7] homozygous for a novel *SDHD* c.[275A > G];[275A > G];p.[(Asp92Gly)];[(Asp92Gly)] substitution, presenting with cardiomyopathy in utero. He developed cardiopulmonary insufficiency rapidly after birth, dying on day 1 of life. Subsequent analysis of respiratory chain function in patient muscle homogenate revealed a marked defect in complex II activity.

The four affected individuals described here show phenotypic overlap with both these individuals (Table 1). Our study extends the clinical spectrum and highlights the wide range of phenotypical features and severity across affected individuals, even those with the same *SDHD* genotype (Table 1). Hypertrichosis, a recognised feature of some forms of mitochondrial disease (most notably SURF1-Leigh syndrome [16]), was a noted feature in two Palestinian children. Hirschsprung disease diagnosed in a single affected individual (V:2) has not been previously reported in association with *SDHD* variants, and it remains unclear whether this is an associated or unrelated feature. Neurodevelopmental regression is a common characteristic of mitochondrial disease, particularly during physiologic stress through intercurrent infection, prolonged fasting or dehydration [17]. It is thus unsurprising that this appears to be a common feature of complex II deficiency due to biallelic *SDHD* variants (Table 1). An accurate molecular diagnosis for complex II deficient patients would support avoidance of prolonged fasting and dehydration.

Homology modelling of the two putative pathogenic *SDHD* missense variants thus far associated with isolated autosomal recessive mitochondrial complex II deficiency [c.[205G > A];p.(Glu69Lys) and c.[275A > G];p.(Asp92Gly)] predicts disruption of non-covalent bonds between transmembrane helices and changes to complex II positioning in the inner mitochondrial membrane as likely outcomes (Supplementary Fig. S1a). In addition to their role in primary mitochondrial disease, heterozygous germline variants in other complex II subunits and assembly factors (including *SDHA*, *SDHB*, *SDHC*, *SDHD* and *SDHAF2)* are associated with paragangliomas, phaeochromocytomas and gastrointestinal stromal tumours [3]. None of the three *SDHD* variants associated with mitochondrial complex II deficiency have been previously linked to tumourigenesis, including in this extended Palestinian family, although a Dutch founder familial paraganglioma *SDHD* variant c.274G > T; p.(Asp92Tyr) has been described [18]. Additionally, *SDHA* and *SDHB* variants have been associated with both mitochondrial complex II deficiency in biallelic form, and hereditary cancer susceptibility in monoallelic form [5, 19]. Therefore routine surveillance of heterozygous *SDHD* carriers is suggested for early detection of paragangliomas and phaeochromocytomas and appropriate intervention. Together the data presented here consolidate biallelic *SDHD* variants as a cause of mitochondrial disease due to mitochondrial complex II malfunction, and extend the variable associated clinical features.

## Supplementary information

Supplemental figures S1, S2A and S2B
