# Supplementary material for: Consolidating biallelic SDHD variants as a cause of mitochondrial complex II deficiency
Source: Eur J Hum Genet. 2021 May 20;29(10):1570–6. doi: 10.1038/s41431-021-00887-w (PMC8484551; doi:10.1038/s41431-021-00887-w)
Supplement: Supplementary file 1 — Supplemental figures S1, S2A and S2B [file 41431_2021_887_MOESM1_ESM.docx]

**Consolidating biallelic *SDHD* variants as a cause of mitochondrial complex II deficiency**

Siying Lin^1$^, James Fasham^1,2$^, Fida’ Al-Hijawi^3$^, Nouar Qutob^4^, Adam Gunning^1^, Joseph S Leslie^1^, Lucy McGavin^5^, Nishanka Ubeyratna^1^, Wisam Baker^6^, Ramez Zeid^7^, Peter D Turnpenny^2^, Andrew H Crosby^1^, Emma L Baple^1,2,*^ Reham Khalaf-Nazzal^7,*^

**Affiliations**

1. RILD Wellcome Wolfson Centre, University of Exeter Medical School, Royal Devon & Exeter NHS Foundation Trust, Barrack Road, Exeter, UK
2. Peninsula Clinical Genetics, Royal Devon & Exeter Hospital (Heavitree), Gladstone Road, Exeter, UK
3. Paediatrics’ Community Outpatient Clinics, Palestinian Ministry of Health, Jenin, Palestine
4. Department of Health Sciences, Faculty of Graduate Studies, Arab American University of Palestine, Ramallah, Palestine
5. University Hospitals Plymouth NHS Trust, Derriford Road, Crownhill, Plymouth, UK
6. Paediatrics Department, Dr. Khalil Suleiman Government Hospital, Jenin, Palestine
7. Biomedical Sciences Unit and Faculty of Dentistry, Arab American University of Palestine, Jenin, Palestine

$. These authors contributed equally

**Supplemental Figure S1: Localisation informs pathogenic mechanism of SDHD missense variants associated with complex II deficiency.**

**A. Human complex II structural homology model** from an experimentally-derived porcine structure [3abv]. IMM: Inner mitochondrial membrane, IMS: Inter-membrane space, SDHA; SDHB; SDHC; SDHD: Succinate dehydrogenase complex, subunits A/B/C/D. *yellow dashed lines*: polar bonds with the potential to be disrupted

Modelling of p.(Glu69Lys)(E69K) *[lower inset]*, predicts disruption of a hydrogen bond with Gln109 due to a reversal of the negative charge present on glutamic acid, with possible resulting structural instability of the tertiary structure and resultant effects on protein stability and abundance.

Alston *et al.* previously concluded that the p.(Asp92Gly)(D92G) variant *[upper inset]* may destabilise the alpha helix or be important in “capping the helical dipole”. The latter conclusion is consistent with our results: this highly conserved charged residue appears to lie near or at the IMM/IMS interface in this region **B. SDHD Amino acid alignment.** The position of Glu69 and Asp92 are shown with arrowheads. If the p.(Asp92Gly) residue has a membrane anchoring role, it is likely that the loss of this charge would greatly impair this function, [scale from hydrophobic (red), uncharged (purple) to charged (blue)].

**A**.
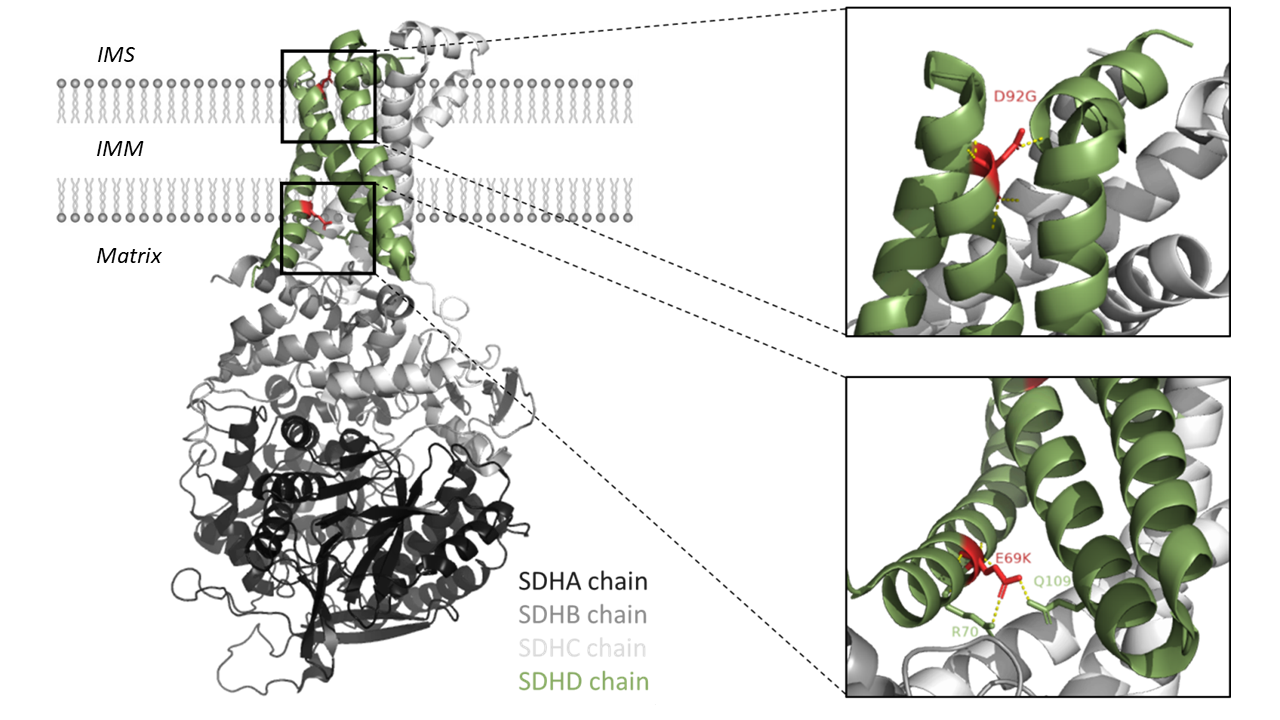


**B.** 62 70 80 90 100

| | | | |

Human

Chimpanzee

Macaque

Dog

Cow

Mouse

Rat

Chicken

Zebrafish

Frog

**Supplemental Figure S2A: Likely benign SDHD missense variants.**

The p.(Ala90Val) and p.(Tyr93Cys) (bright green) amino acid substitutions**,** defined as likely benign by their presence in more than 50 individuals in gnomAD databases (v2.1.1 and v3)**,** are shown to be in close proximity to the pathogenic variant p.(Asp92Gly) (red), but have different predicted effects, importantly neither affect the solitary charged residue.

**A.**


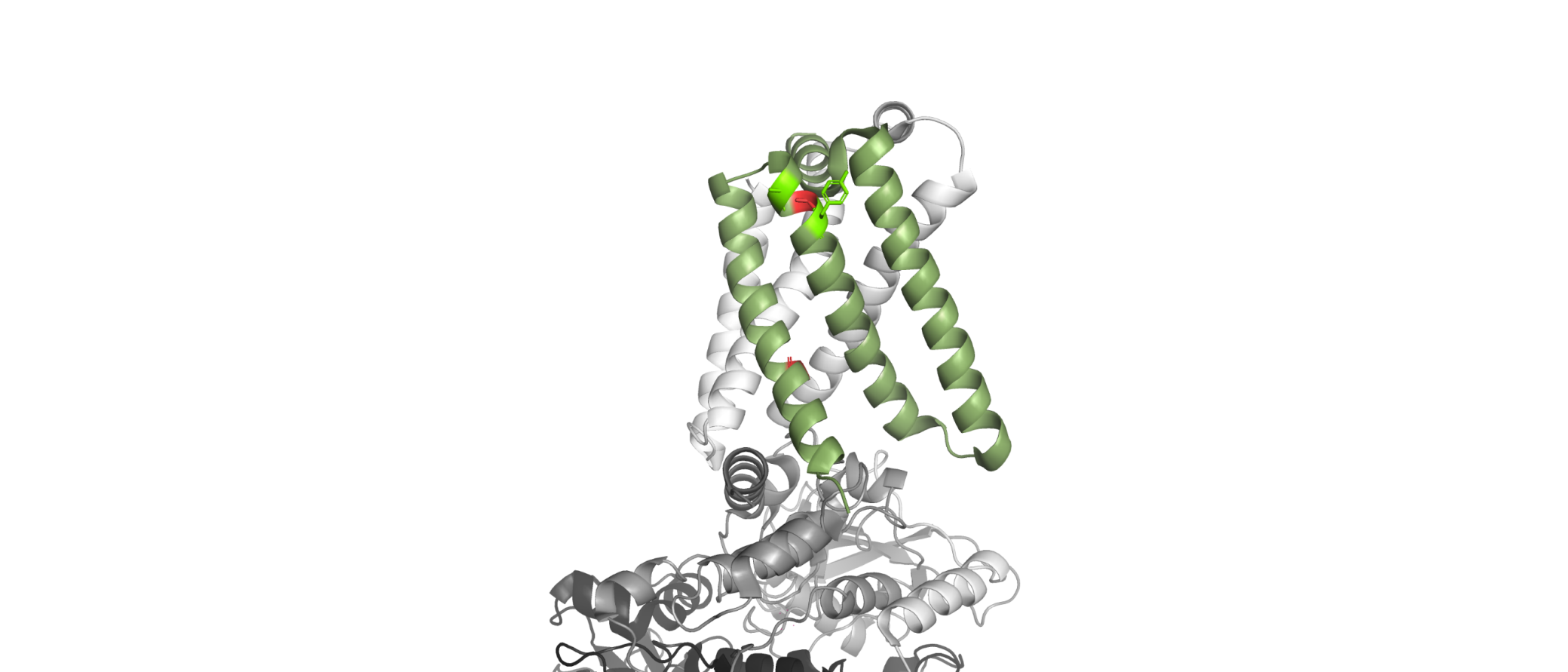


**Supplemental Figure S2B:** **Germline missense SDHD variants associated with hereditary paraganglioma – phaeochromocytoma syndrome**, classified as pathogenic in HGMDPro and ClinVar (25 in total), show no obvious localisation or pattern of distribution.


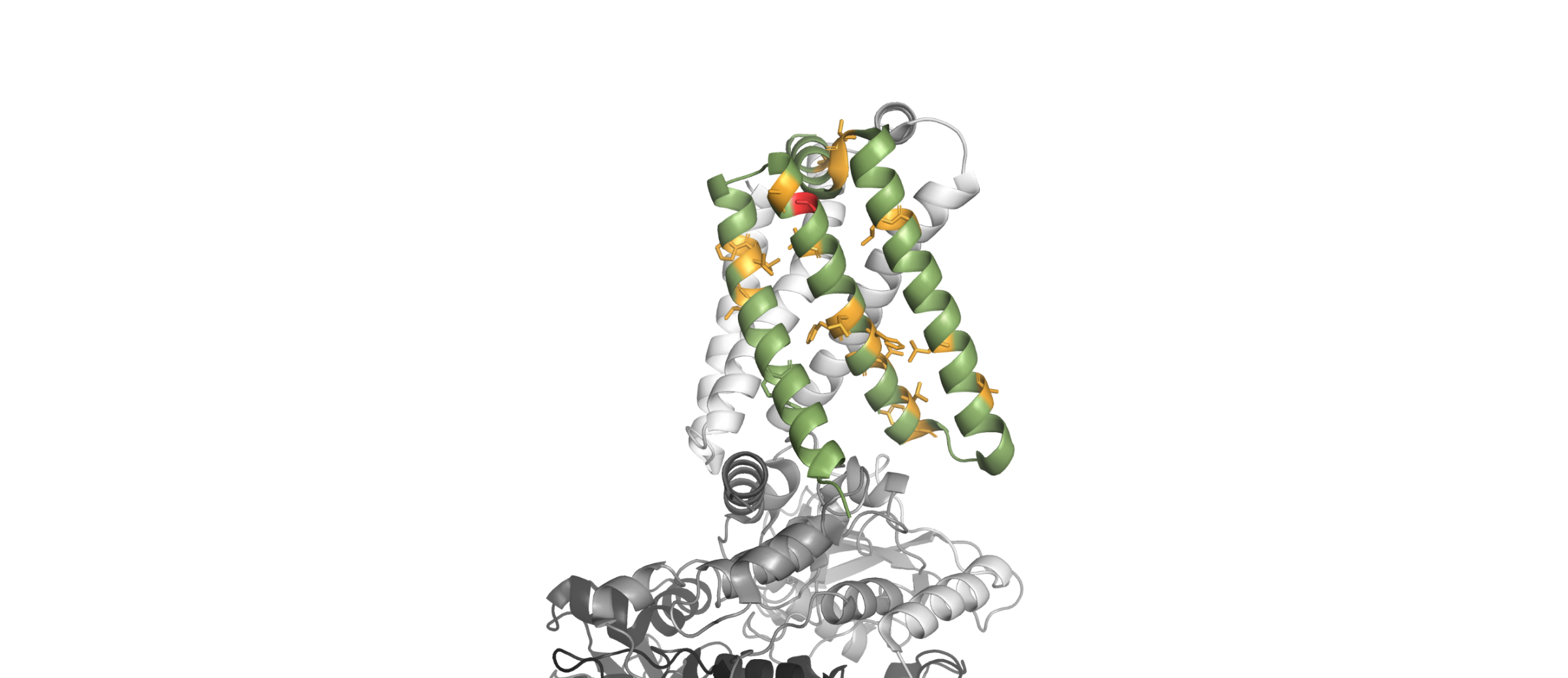
**B.**

| Residue | Name | Condition | Variant Classification |
| --- | --- | --- | --- |
| *Model starts at residue 59* | |  |  |
| 70 | NM_003002.4(SDHD):c.209G>C (p.Arg70Thr) | Paraganglioma | ClinVar Likely Pathogenic {Burnichon, 2009 #1} |
| 70 | NM_003002.4(SDHD):c.209G>T (p.Arg70Met) | Paraganglioma | ClinVar Likely Pathogenic {Burnichon, 2009 #1} |
| 70 | NM_003002.4(SDHD):c.210G>T (p.Arg70Ser) | Paraganglioma / phaeochromocytoma | {Cascón, 2009 #2} |
| 77 | NM_003002.4(SDHD):c.230T>G (p.Leu77Arg) | Paraganglioma | {Neumann, 2009 #3} |
| 79 | NM_003002.4(SDHD):c.236T>G (p.Leu79Arg) | Paraganglioma / phaeochromocytoma | {Prasad, 2014 #4} |
| 80 | NM_003002.4(SDHD):c.239T>G (p.Leu80Arg) | Paraganglioma | {Burnichon, 2009 #1} |
| 81 | NM_003002.4(SDHD):c.242C>T (p.Pro81Leu) | Paraganglioma / phaeochromocytoma / other | ClinVar Pathogenic {Baysal, 2000 #5} |
| 90 | NM_003002.4(SDHD):c.268G>A (p.Ala90Thr) | Phaeochromocytoma | {Cascon, 2004 #6} |
| 92 | NM_003002.4(SDHD):c.274G>T (p.Asp92Tyr) | Paraganglioma / phaeochromocytoma / other | ClinVar Pathogenic {Baysal, 2000 #5} |
| 95 | NM_003002.4(SDHD):c.284T>G (p.Leu95Arg) | Paraganglioma | {Heesterman, 2018 #7} |
| 95 | NM_003002.4(SDHD):c.284T>C (p.Leu95Pro) | Paraganglioma / phaeochromocytoma | ClinVar Pathogenic {Taschner, 2001 #8} |
| 101 | NM_003002.4(SDHD):c.302T>C (p.Leu101Pro) | Paraganglioma | {Neumann, 2009 #3} |
| 102 | NM_003002.4(SDHD):c.304C>T (p.His102Tyr) | Paraganglioma / phaeochromocytoma / other | ClinVar Likely Pathogenic {Piccini, 2012 #9} |
| 102 | NM_003002.4(SDHD):c.304C>A (p.His102Asn) | Paraganglioma / phaeochromocytoma / other | ClinVar Pathogenic/Likely Pathogenic {LaDuca, 2017 #10} |
| 102 | NM_003002.4(SDHD):c.305A>T (p.His102Leu) | Paraganglioma / phaeochromocytoma / other | ClinVar Pathogenic/Likely Pathogenic {Baysal, 2000 #5} |
| 104 | NM_003002.4(SDHD):c.311A>C (p.His104Pro) | Paraganglioma | {Zheng, 2012 #11} |
| 106 | NM_003002.4(SDHD):c.317G>A (p.Gly106Asp) | Paraganglioma | {Ogawa, 2006 #12} |
| 106 | NM_003002.4(SDHD):c.317G>T (p.Gly106Val) | Paraganglioma | {Neumann, 2009 #3} |
| 107 | NM_003002.4(SDHD): c.320T>C (p.Leu107Pro) | Cardiac paraganglioma | {Otani, 2017 #13} |
| 111 | NM_003002.4(SDHD):c.331G>A (p.Val111Ile) | Paraganglioma | {Isobe, 2007 #14} |
| 114 | NM_003002.4(SDHD):c.340T>A (p.Tyr114Asn) | Hereditary cancer-predisposing syndrome | ClinVar Likely Pathogenic {Milunsky, 2001 #15} |
| 114 | NM_003002.4(SDHD):c.341A>G (p.Tyr114Cys) | Paraganglioma / phaeochromocytoma / other | ClinVar pathogenic |
| 123 | NM_003002.4(SDHD):c.367G>A (p.Ala123Thr) | Paraganglioma | {Neumann, 2009 #3} |
| 128 | NM_003002.4(SDHD):c.383T>C (p.Leu128Pro) | Paraganglioma | {Dreijerink, 2019 #16} |
| 138 | NM_003002.4(SDHD):c.412G>A (p.Gly138Arg) | Paraganglioma / phaeochromocytoma / other | ClinVar Likely Pathogenic {Turner, 2019 #17} |
| 139 | NM_003002.4(SDHD):c.416T>C (p.Leu139Pro) | Paraganglioma / phaeochromocytoma | ClinVar Pathogenic {Taschner, 2001 #8} |
| 147 | NM_003002.4(SDHD):c.439G>A (p.Val147Met) | Paraganglioma | {Alataki, 2010 #18} |
| 148 | NM_003002.4(SDHD):c.443G>A (p.Gly148Asp) | Paraganglioma / phaeochromocytoma | ClinVar Likely Pathogenic {Benn, 2006 #19} |
| 148 | NM_003002.4(SDHD):c.443G>T (p.Gly148Val) | Paraganglioma | ClinVar Likely Pathogenic {Neumann, 2004 #20} |
| 156 | NM_003002.4(SDHD):c.467T>C (p.Leu156Pro) | Paraganglioma | {Piccini, 2012 #9} |

**Table: SDHD Variants causing paraganglioma or phaeochromocytoma included in Supplemental Figure S2B**
 Including all variants from residue 59 onwards classified as likely Pathogenic or pathogenic on 15/02/2021

## **Supplemental References**

1. Burnichon N, Rohmer V, Amar L, Herman P, Leboulleux S, Darrouzet V et al. The Succinate Dehydrogenase Genetic Testing in a Large Prospective Series of Patients with Paragangliomas. The Journal of Clinical Endocrinology & Metabolism. 2009;94(8):2817-2827.

2. Cascón A, Pita G, Burnichon N, Landa I, López-Jiménez E, Montero-Conde C et al. Genetics of Pheochromocytoma and Paraganglioma in Spanish Patients. The Journal of Clinical Endocrinology & Metabolism. 2009;94(5):1701-1705.

3. Neumann H, Erlic Z, Boedeker C, Rybicki L, Robledo M, Hermsen M et al. Clinical Predictors for Germline Mutations in Head and Neck Paraganglioma Patients: Cost Reduction Strategy in Genetic Diagnostic Process as Fall-Out. Cancer Research. 2009;69(8):3650-3656.

4. Prasad C, Oakley G, Yip L, Coyne C, Rangaswamy B, Dixit S. A novel mutation in the succinate dehydrogenase subunit D gene in siblings with the hereditary paraganglioma–pheochromocytoma syndrome. SAGE Open Medical Case Reports. 2014;2:2050313X1455352.

5. Baysal B. Mutations in SDHD, a Mitochondrial Complex II Gene, in Hereditary Paraganglioma. Science. 2000;287(5454):848-851.

6. Cascon A. Genetic and epigenetic profile of sporadic pheochromocytomas. Journal of Medical Genetics. 2004;41(3):30e-30.

7. Heesterman B, de Pont L, van der Mey A, Bayley J, Corssmit E, Hes F et al. Clinical progression and metachronous paragangliomas in a large cohort of SDHD germline variant carriers. European Journal of Human Genetics. 2018;26(9):1339-1347.

8. Taschner P, Jansen J, Baysal B, Bosch A, Rosenberg E, Bröcker-Vriends A et al. Nearly all hereditary paragangliomas in The Netherlands are caused by two founder mutations in theSDHDgene. Genes, Chromosomes and Cancer. 2001;31(3):274-281.

9. Piccini V, Rapizzi E, Bacca A, Di Trapani G, Pulli R, Giachè V et al. Head and neck paragangliomas: genetic spectrum and clinical variability in 79 consecutive patients. Endocrine-Related Cancer. 2012;19(2):149-155.

10. LaDuca H, Farwell K, Vuong H, Lu H, Mu W, Shahmirzadi L et al. Exome sequencing covers >98% of mutations identified on targeted next generation sequencing panels. PLOS ONE. 2017;12(2):e0170843.

11. Zheng X, Wei S, Yu Y, Xia T, Zhao J, Gao S et al. Genetic and clinical characteristics of head and neck paragangliomas in a chinese population. The Laryngoscope. 2012;122(8):1761-1766.

12. Ogawa K, Shiga K, Saijo S, Ogawa T, Kimura N, Horii A. A novel G106D alteration of theSDHDgene in a pedigree with familial paraganglioma. American Journal of Medical Genetics Part A. 2006;140A(22):2441-2446.

13. Otani N, Sugano K, Inami S, Amano H, Arikawa T, Saito S et al. Cardiac paraganglioma with a novel germline mutation of succinate dehydrogenase gene D. Japanese Journal of Clinical Oncology. 2017;47(12):1193-1197.

14. Isobe K, Minowada S, Tatsuno I, Suzukawa K, Nissato S, Nanmoku T et al. Novel Germline Mutations in the SDHB and SDHD Genes in Japanese Pheochromocytomas. Hormone Research in Paediatrics. 2007;68(2):68-71.

15. Milunsky J, Maher T, Michels V, Milunsky A. Novel mutations and the emergence of a common mutation in theSDHD gene causing familial paraganglioma. American Journal of Medical Genetics. 2001;100(4):311-314.

16. Dreijerink K, Rijken J, Compaijen C, Timmers H, van der Horst-Schrivers A, van Leeuwaarde R et al. Biochemically silent sympathetic Paraganglioma, Pheochromocytoma or Metastatic Disease in SDHD mutation carriers. The Journal of Clinical Endocrinology & Metabolism. 2019;.

17. Turner S, Rao S, Morgan R, Vnencak-Jones C, Wiesner G. The impact of variant classification on the clinical management of hereditary cancer syndromes. Genetics in Medicine. 2018;21(2):426-430.

18. Alataki D, Triantafyllidis A, Gaal J, Rodiou C, Vouros J, Papathanasiou A et al. A non-catecholamine-producing sympathetic paraganglioma of the spermatic cord: the importance of performing candidate gene mutation analysis. Virchows Archiv. 2010;457(5):619-622.

19. Benn D, Gimenez-Roqueplo A, Reilly J, Bertherat J, Burgess J, Byth K et al. Clinical Presentation and Penetrance of Pheochromocytoma/Paraganglioma Syndromes. The Journal of Clinical Endocrinology & Metabolism. 2006;91(3):827-836.

20. Neumann H. Distinct Clinical Features of Paraganglioma Syndromes Associated With SDHB and SDHD Gene Mutations. JAMA. 2004;292(8):943.
